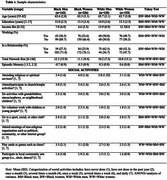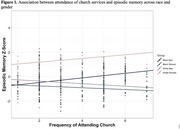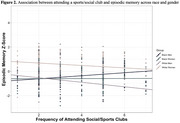# Intersectional race and gender differences in the forms of social engagement that matter most for cognitive health

**DOI:** 10.1002/alz70860_105098

**Published:** 2025-12-23

**Authors:** Kiana A. Scambray, Emily P. Morris, Jordan D. Palms, Robrielle M. Pierce, Monica E. Walters, Lauren Taylor, Sofia Lomba, Noah Green, Ketlyne Sol, Laura B. Zahodne

**Affiliations:** ^1^ University of Michigan, Ann Arbor, MI, USA

## Abstract

**Background:**

Greater social engagement has been associated with better cognitive health, but few studies have considered how associations involving specific activities may differ across the intersection of race and gender. Indeed, prior work grounded in a socioecological lens have identified gender norms/roles, culture, and sociodemographics as influential factors on how older men and women engage socially, yet few have considered how these factors intersect (Ong et al., 2024). Examining multiple forms of social engagement and clarifying group‐specific links to cognition may guide more targeted social interventions to mitigate dementia risk and advance health equity. This study evaluated associations between various social activities and episodic memory through an intersectional lens.

**Method:**

Participants included Black men (*n* = 142), Black women (*n* = 226), White men (*n* = 120), and White women (*n* = 153) from the Michigan Cognitive Aging Project. Social engagement was operationalized as frequency of participation in eight activities such as attending religious services, activities/volunteer work with children, and playing games with a group (Table 1). Episodic memory was a z‐score composite of word list learning, story memory, and visual memory tests. ANOVAs and Tukey tests assessed group differences across race and gender. Four multiple regression models, stratified by race and gender, assessed unique associations between all social engagement measures and episodic memory adjusting for education, income, work status, marital status, and social network size.

**Result:**

Among Black men, attending religious services (B=0.19, *p* = 0.049) and playing card games (B=0.21, *p* = 0.01) more frequently were associated with better memory. Among White men, more frequent attendance to a sport/social club was associated with worse memory (B=‐0.19, *p* = 0.04). There were no significant associations between social engagement and memory among Black or White women.

**Conclusion:**

This study supports the importance of participating in social engagement, particularly for Black men. Findings also highlight the value of taking an intersectional lens and considering multiple forms of social engagement, as activities may be differentially associated with cognitive health across race and gender. Longitudinal and interventional investigations are needed to better understand implications of social activities and their optimal frequency of engagement on cognitive health for individualized dementia prevention.